# What the papers say

**DOI:** 10.1093/jhps/hnx024

**Published:** 2017-06-01

**Authors:** Ajay Malviya

The *Journal of Hip Preservation Surgery* (*JHPS*) is not the only place where work in the field of hip preservation may be published. Although our aim is to offer the best of the best, we continue to be fascinated by work that finds its way into journals other than our own. There is much to learn from it so *JHPS* has selected six recent and topical articles for those who seek a brief summary of what is taking place in our ever-fascinating world of hip preservation. What you see here are the mildly edited abstracts of the original articles, to give them what *JHPS* hopes is a more readable feel. If you are pushed for time, what follows should take you no more than 10 min to read. So here goes …

## PAIN MANAGEMENT STRATEGIES AFTER HIP ARTHROSCOPY

Post-operative pain management can be an issue after hip arthroscopy and each of us endeavours to provide the best regime for the patients. Kahlenberg *et al.* [1] from North Western University, Chicago set up a randomised controlled trial to determine the efficacy of pre-operative Celecoxib for early post-operative pain management in hip arthroscopy in terms of reduction in pain, narcotic requirement and early discharge.

Ninety-eight patients were randomised to either the Celecoxib group (*n* = 50) or the placebo group (*n* = 48). *A priori* power analysis was done, set to detect a difference of 0.50 on the visual analogue scale (VAS), based on the senior author's preference. The number of patients planned for recruitment was rounded up to 100 to allow for flexibility in the study. Inclusion criteria were any patient at least 18 years old who underwent hip arthroscopy surgery performed by the senior author. All patients had less than Tönnis grade two arthritis. Exclusion criteria were allergy to sulpha-based drugs, prior adverse reaction to celecoxib, or patients who were on chronic narcotics for whom alternative pain management regimens were arranged before surgery. Randomisation was performed on one:one basis in blocks of 10 using sealed envelopes stating celecoxib or placebo. One hour before surgery, all patients received either 400 mg celecoxib or placebo. Patients were evaluated using a VAS pre-operatively, immediately post-operatively, and at 1 and 2 h post-operatively. Time from the operating room to ‘ready for discharge’ and number of morphine equivalents of narcotic medication required in the post-anaesthesia care unit were recorded.

Age and pre-operative VAS were similar between the celecoxib and placebo control group, with average ages of 34.2 and 35.8 (*P* = 0.27) years and pre-operative VAS of 2.1 and 2.3 (*P* = 0.29), respectively. The celecoxib group had 26 females and 24 males, whereas the placebo group had 29 females and 19 males (*P* = 0.42). The most common surgical procedures were labral repair (31 patients in the celecoxib group and 29 patients in the placebo group), and labral repair with acetabular osteoplasty (13 patients in the celecoxib group and 11 patients in the placebo group). There were no significant differences in procedures performed between the two groups (*P* > 0.05). At 1 h post-operatively, patients who received celecoxib had a lower pain score that was statistically significant compared with the placebo group (4.6 vs. 5.4, *P* = 0.03). There was a significant difference in discharge time between patients who received celecoxib and the control group (152.9 vs. 172.9 min, *P* = 0.04). There was no significant difference found in morphine equivalents consumed in the post-anaesthesia care unit between the two groups (15.3 vs. 15.4, *P* = 0.48).

The authors concluded that a pre-operative dose of 400 mg of celecoxib led to statistically significantly reduced patient-reported pain on the VAS in the acute post-operative period after hip arthroscopy surgery, though the difference is not likely clinically significant. There was a significantly shorter time to discharge in patients who received celecoxib versus placebo.

## ROLE OF HIP ARTHROSCOPY IN PAINFUL HIPS WITH NORMAL IMAGING

Many practitioners have encountered patients with hip pain that can be a diagnostic dilemma. Australian researchers [2] set out to explore the role of hip arthroscopy in this group of complex patients with groin, hip and pelvic pain but normal findings on magnetic resonance imaging (MRI) and minimal changes on X-ray. They looked at the arthroscopic findings of patients who have had hip pain and a positive response to an intra-articular anaesthetic but have non-contributory imaging. They hypothesised that standard MRIs were missing significant pathology and if there was a response to intra-articular local anaesthesia, it was likely that there will be a pathology found during arthroscopy.

They retrospectively reviewed all hip arthroscopies performed from March 2011 to January 2015 by two orthopaedic surgeons specialising in hip arthroscopy to identify patients with clinically suspected intra-articular hip pathology despite a normal imaging. Fifty-three hip arthroscopies performed in 51 patients met the inclusion criteria from a total of 1348 hip arthroscopies performed over a 46-month period. All but one of the 53 (98%) hips had arthroscopically confirmed pathology. Mean patient age was 32.5 years (15–67) with 40 (78%) females and 11 (22%) males. 92.5% of the hips (49/53) were FADIR (flexion, adduction and internal rotation) positive on clinical examination, giving this test a positive predictive value of 98% (95% CI: 89.31–99.67%) for intra-articular pathology.

In conclusion, in patients with a normal MRI without contrast and a positive response (relief of pain) to an intra-articular injection that failed conservative management, there is a 98% chance of intra-articular hip pathology being discovered on hip arthroscopy.

## DEFINING MODES OF FAILURE AFTER JOINT-PRESERVING SURGERY OF THE HIP

Joint-preserving surgery of the hip (JPSH) has evolved considerably and there are a number of different factors that potentially can lead to failure. Consequently, it is of interest to assess the various modes of failure in order to continue to identify best practice and the indications for these procedures.

Beaulé *et al.* [3] from Ottawa, Canada using a retrospective observational study design, reviewed 1013 patients who had undergone JPSH by a single surgeon between 2005 and 2015. There were 509 men and 504 women with a mean age of 39 years (16–78). Of the 1013 operations, 783 were arthroscopies, 122 surgical dislocations and 108 peri-acetabular osteotomies (PAO). They analysed the overall failure rates and modes of failure. Re-operations were categorised into four groups: Mode 1 was arthritis progression or organ failure leading to total hip arthroplasty (THA); Mode 2 was an Incorrect diagnosis/procedure; Mode 3 resulted from malcorrection of femur (type A), acetabulum (type B) or labrum (type C) and Mode 4 resulted from an unintended consequence of the initial surgical intervention.

At a mean follow-up of 2.5 years, there had been 104 re-operations (10.2%) with a mean patient age of 35.5 years (17–64). There were 64 Mode 1 failures (6.3%) at a mean of 3.2 years following JPSH with a mean patient age of 46.8 years. There were 17 Mode 2 failures (1.7%) at a mean of 2.2 years post-JPSH with a mean patient age of 28.9 years (2% scopes; 1% surgical dislocations). There were 19 Mode 3 failures (1.9%) at a mean of 2.0 years post-JPSH, with a mean patient age of 29.9 years (2% scopes; 2% surgical dislocations; 5% PAO). There were four Mode four failures (0.4%) at a mean of 1.8 years post-JPSH with a mean patient age of 31.5 years. Using the modified Dindo–Clavien classification system, the overall complication rate among JPSHs was 4.2%.

The authors concluded that while defining the overall re-operation and complication rates, it is important to define the safety and effectiveness of JPSH. Standardisation of the modes of failure may help identify the best practice. Application of these modes to large clinical series, such as registries, will assist in further establishing how to improve the efficacy of JPSH.

## ROLE OF HIP ARTHROSCOPY IN REALIGNMENT SURGERY FOR STABLE SLIPPED CAPITAL FEMORAL EPIPHYSIS

Surgeons are pushing the boundaries of hip arthroscopic intervention and Roos *et al.* [4] from Passo Fundo, Brazil have extended its role in deformity correction in slipped capital femoral epiphysis (SCFE). This study aimed to evaluate the clinical and radiographic outcomes, as well as the complications of arthroscopic subcapital realignment osteotomy in chronic and stable SCFE.

Between June 2012 and December 2014, seven patients underwent arthroscopic subcapital realignment osteotomy in chronic and stable SCFE. The mean age was 11 years and 4 months, and the mean follow-up period was 16.5 months (6–36). Clinical results were evaluated using the Modified Harris Hip Score (MHHS), which was measured pre- and post-operatively. Radiographs were evaluated using the Southwick quantitative classification and the epiphysis–diaphysis angle (pre- and post-operatively).

The mean pre-operative MHHS was 35.8 points, and 97.5 points post-operatively (*P* < 0.05). Radiographically, five patients were classified as Southwick classification grade II and two as grade III. The mean correction of the epiphysis–diaphysis angle was 40°. No immediate post- operatively complications were observed. One patient presented femoral head avascular necrosis, without collapse or chondrolysis at the most recent follow-up (22 months).

The authors concluded that their arthroscopic technique for subcapital realignment osteotomy in chronic and stable SCFE showed satisfactory short-term clinical and radiographic outcome.

## DOES PERIACETABULAR OSTEOTOMY COMPROMISE THE BONE BIRTH CANAL?

Traditionally, it is believed that periacetabular osteotomy (PAO) would not compromise the pelvic dimensions and would not be a relative indication for Caesarean section. Japanese researchers [5] have undertaken a three-dimensional computed tomography analysis on bony birth canal after bilateral periacetabular osteotomy. They have traditionally performed the curved periacetabular osteotomy (CPO) for developmental dysplasia of the hip. CPO requires osteotomy of the medial wall of the acetabulum, which may cause narrowing of the bony birth canal and this step may result in increased risk of caesarean delivery.

They analysed the narrowest part of the bony birth canal using three-dimensional computed tomography (3 D-CT) before and after bilateral CPO. The study included 29 cases of bilateral CPO performed between February 2007 and March 2014 in which both pre- and post-operative 3 D-CT were available. Transverse diameters of the pelvic inlet, contraction, outlet, expansion and teardrop were analysed. Among them, the narrowest part of the bony birth canal was investigated, which if smaller than the normal lower threshold value for vaginal delivery (95 mm) was considered as a risk for Caesarean delivery.

The transverse diameters of both pelvic expansion and teardrop significantly decreased after CPO (both *P* < 0.01), while other diameters showed no significant changes. Amongst these two diameters, the narrowest diameter of the bony birth canal was the pelvic teardrop in all 29 cases. That in 24 patients (82.8%) was greater than 95 mm, while that in five patients (17.2%) showed less than 95 mm.

The authors concluded that based on 3 D-CT analysis, the narrowest part of the bony birth canal after bilateral CPO was the pelvic teardrop and because in more than 80% the diameter was greater than 95 mm; there was no real contraindication for vaginal delivery in the majority.

## ROLE OF PAO VERSUS ACETABULAR RIM TRIMMING IN THE TREATMENT OF IMPINGEMENT CAUSED BY ACETABULAR RETROVERSION

Acetabular retroversion can cause femoroacetabular impingement leading to hip pain and osteoarthritis. It can be treated by anteverting PAO or acetabular rim trimming with refixation of the labrum. There is increasing evidence that acetabular retroversion is a rotational abnormality of the entire hemipelvis and not a focal overgrowth of the anterior acetabular wall, which favours an anteverting PAO. However, it is unknown if this larger procedure would be beneficial in terms of survivorship and improvement in functional outcome in a midterm follow-up compared with rim trimming.

Zurmuhle *et al.* [6] from Bern, Switzerland have performed a retrospective, comparative study evaluating the midterm survivorship of two matched patient groups with symptomatic acetabular retroversion undergoing either anteverting PAO or acetabular rim trimming through a surgical hip dislocation. Acetabular retroversion was defined by concomitant positive crossover, posterior wall and ischial spine signs.

A total of 279 hips underwent a surgical intervention for acetabular retroversion at Bern University hospital between 1997 and 2012 (166 periacetabular osteotomies, 113 rim trimmings through surgical hip dislocation). A total of 99 patients (60%) were excluded from the PAO group and 56 patients (50%) from the rim-trimming group because they had pre-specified conditions for exclusion, for purposes of matching, deficient records or the patient declined or was lost to follow-up.

This left 67 hips (57 patients) that underwent anteverting PAO and 57 hips (52 patients) that had acetabular rim trimming. The two groups did not differ in terms of age, sex, body mass index, pre-operative ROM, pre-operative Merle d'Aubigné-Postel score, radiographic morphology of the acetabulum (except total and anterior acetabular coverage), alpha angle, Tönnis grade of osteoarthritis and labral and chondral lesions on the preoperative MRI. A minimum follow-up of 2 years was required for this study. Failures were included at any time. The median follow-up for the anteverting PAO group was 9.5 years (range, 2–17.4 years) and 6.8 years (range, 2.2–10.5 years) for the rim trimming group (*P* < 0.001). Kaplan–Meier survivorship analysis was performed using the following endpoints at 5 and 10 years: THA, radiographic progression of osteoarthritis by one Tönnis grade and/or Merle d'Aubigné-Postel score < 15 points.

Although the 5-year survivorship of the two groups was not different with the numbers available; 86% for anteverting PAO versus 86% for acetabular rim trimming, the study found increased survivorship at 10 years in hips undergoing anteverting PAO for acetabular retroversion (79%) compared with acetabular rim trimming (23%) at 10 years (*P* < 0.001). The drop in the survivorship curve for the acetabular rim trimming through surgical hip dislocation group started at year six. The main reason for failure was a decreased Merle d'Aubigné score.

The authors concluded that anteverting PAO may be the more appropriate treatment for hips with substantial acetabular retroversion. This may be the result of reduction of an already smaller lunate surface of hips with acetabular retroversion through rim trimming. However, rim trimming may still benefit hips with acetabular retroversion in which only one or two of the three signs are positive. The authors recommended that future randomised studies should compare these treatments. 

## CONFLICT OF INTEREST STATEMENT

None declared.
